# Regulation of ASIC channels by a stomatin/STOML3 complex located in a mobile vesicle pool in sensory neurons

**DOI:** 10.1098/rsob.120096

**Published:** 2012-06

**Authors:** Liudmila Lapatsina, Julia A. Jira, Ewan St. J. Smith, Kate Poole, Alexey Kozlenkov, Daniel Bilbao, Gary R. Lewin, Paul A. Heppenstall

**Affiliations:** 1 Department of Neuroscience, Max-Delbrück Center for Molecular Medicine, Robert-Rössle-Strasse 10, 13092 Berlin-Buch, Germany; 2 Department of Anesthesiology, Campus Benjamin Franklin, Charité-Universitätsmedizin, Hindenburgdamm 30, 12200 Berlin, Germany; 3 Mouse Biology Unit, European Molecular Biology Laboratory (EMBL), Via Ramarini 32, 00015 Montorotondo, Italy

**Keywords:** ion channel, mechanotransduction, peripheral sensory neurons, stomatin-like proteins

## Abstract

A complex of stomatin-family proteins and acid-sensing (proton-gated) ion channel (ASIC) family members participate in sensory transduction in invertebrates and vertebrates. Here, we have examined the role of the stomatin-family protein stomatin-like protein-3 (STOML3) in this process. We demonstrate that STOML3 interacts with stomatin and ASIC subunits and that this occurs in a highly mobile vesicle pool in dorsal root ganglia (DRG) neurons and Chinese hamster ovary cells. We identify a hydrophobic region in the N-terminus of STOML3 that is required for vesicular localization of STOML3 and regulates physical and functional interaction with ASICs. We further characterize STOML3-containing vesicles in DRG neurons and show that they are Rab11-positive, but not part of the early-endosomal, lysosomal or Rab14-dependent biosynthetic compartment. Moreover, uncoupling of vesicles from microtubules leads to incorporation of STOML3 into the plasma membrane and increased acid-gated currents. Thus, STOML3 defines a vesicle pool in which it associates with molecules that have critical roles in sensory transduction. We suggest that the molecular features of this vesicular pool may be characteristic of a ‘transducosome’ in sensory neurons.

## 2. Introduction

Stomatin-like protein-3 (STOML3) is a member of a large protein family characterized by the presence of a stomatin signature domain [1,2]. Proteins containing a stomatin domain are present in most organisms [3,4], and recently the X-ray crystal structure of this domain in PH1511, a protein from the hyperthermophilic archaeon *Pyrococcus horikoshii*, was solved [5]. Although PH1511 is a prokaryotic protein, the amino acid sequence of the stomatin domain is remarkably close to that of its mammalian orthologues. In mammals, the stomatin-domain proteins are almost all integral membrane proteins or closely associated with membranes [2].

STOML3 was first identified in the olfactory epithelium of the mouse [6,7] but, aside from its cellular localization [8], its function there is currently unknown. The major loss-of-function phenotypes demonstrated so far for stomatin-like proteins affect neuronal cells [9–12] and renal podocytes [13,14]. STOML3 and stomatin are expressed by primary sensory neurons of the dorsal root ganglia (DRG) [9,15] and regulate mechanoreceptor sensitivity in mice [9,11]. Similarly, MEC-2, a *Caenorhabditis elegans* stomatin-like protein, is also required for mechanosensitivity and is necessary for the function of MEC-4/MEC-10-containing mechanosensitive channels in the nematode worm [10,12,16–18]. Intriguingly, mammalian orthologues of MEC-4 and MEC-10, the acid-sensing (proton-gated) ion channels ASIC2a/b and ASIC3, have also been implicated in mechanosensation [19–22]: The ASICs can functionally interact with stomatin and STOML3, and such interactions may contribute to the modulation of sensory neuron mechanosensitivity seen after deletion of ASIC genes [9,23]. Furthermore, evidence from targeted deletion of the *STOML3* gene strongly suggests that this protein is an essential regulator of native mechanosensitive ion channels [2,9].

The starting point of the present study was the finding that both stomatin and STOML3 are expressed by sensory neurons and have been shown to have non-redundant functions in these neurons [9,11]. We therefore asked whether stomatin and STOML3 form a complex and regulate ASICs in sensory neurons. We show that STOML3, an interaction partner of both stomatin and ASIC subunits, identifies a highly mobile vesicle pool in both DRG neurons and Chinese hamster ovary (CHO) cells. We demonstrate that an N-terminal hydrophobic region of STOML3 is required for correct localization within the vesicle pool and is necessary for maintaining complex integrity. Moreover, disassociation of vesicles from microtubules promotes plasma membrane fusion of vesicles and concomitant increases in the amplitude of acid-gated membrane currents. Our data suggest that a group of molecules with critical roles in transduction are associated with each other in highly mobile vesicles; we propose that the molecular properties of this vesicle pool are consistent with the idea that it represents a putative ‘transducosome’ in DRG neurons.

## 3. Results

### 3.1. Subcellular localization of stomatin and stomatin-like protein-3

**Figure 1.**
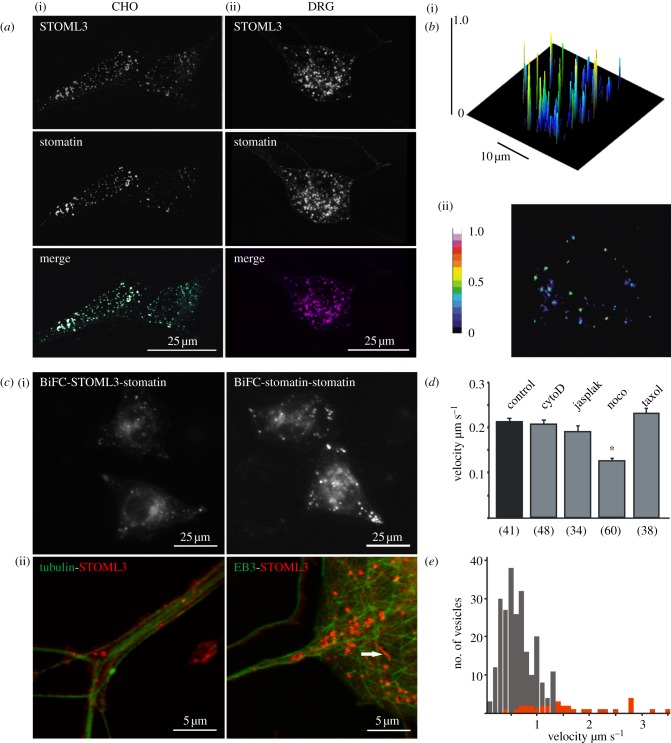
STOML3 and stomatin localize to microtubule-associated vesicles (*a*) STOML3 co-localizes with stomatin in CHO cells (i) and DRG neurons (ii). (*b*) Representative image of FRET efficiency in a single CHO cell co-expressing STOML3-AmCyan1 and EYFP-stomatin. A two-dimensional image (ii) and surface plot with the FRET efficiency projected in colour onto the *z*-axis (i) are shown. (*c*) In CHO cells, stomatin interacts with STOML3 and stomatin itself, as demonstrated using BiFC (i). (ii) Also shown is the co-localization of STOML3 (red) with EB3 or tubulin (green) in DRG neurons. Note that some STOML3 vesicles are very long (arrow). (*d*) Quantification of STOML3-particle motility in CHO cells treated with compounds that destabilize or stabilize actin filaments (cytoD, cytochalasinD; jasplak, jasplakinolide) or microtubules (noco, nocodazole; taxol), respectively. *p < 0.05 versus untreated control. Numbers in parentheses indicate number of cells. (*e*) Frequency distribution of STOML3-positive particle velocities measured for all moving puncta (grey bars) or only for vesicles more than 1 µm in length (red bars). Note that longer vesicles travel with much higher average velocity than the non-selected population.

We used fluorescent-protein-tagged fusion constructs of STOML3 or stomatin to investigate their distribution within CHO cells and DRG neurons. Both STOML3 and stomatin were detected in punctate, vesicular structures distributed throughout the cytoplasm of neuronal and non-neuronal cells (figure 1*a*). When we transfected sensory neurons from Stoml3^−/−^ mice [9] with plasmids encoding fluorescently tagged STOML3, the vesicular localization was indistinguishable from that found in wild-type cells (see the electronic supplementary material, figure S1). For this reason, most subsequent experiments were carried out using sensory neurons from wild-type mice. A closer examination of these puncta revealed that STOML3 was co-localized with stomatin in the majority of vesicles, prompting us to examine whether these proteins interact.

We measured apparent fluorescence resonance energy transfer (FRET) efficiency between STOML3-cyan fluorescent protein (CFP) and stomatin-yellow fluorescent protein (YFP) in CHO cells and observed high levels of FRET within vesicles. Indeed, FRET efficiency approached 50 per cent in many puncta with only a weak signal detected in plasma membrane or cytoplasmic compartments (figure 1*b*). We examined this further using bimolecular fluorescence complementation (BiFC) in which the site of protein–protein interaction can be monitored as a fluorescence signal in cells when two fragments of a fluorescent protein fused to interacting proteins come together [24]. We fused the N- and C-termini of YFP to STOML3 and stomatin, respectively, and could observe a fluorescence signal develop in vesicles in transfected CHO cells indicating BiFC between STOML3 and stomatin. The same technique also revealed self-association between stomatin and stomatin (figure 1*c*). We also observed BiFC signals at the plasma membrane. Taken together, these results indicate that STOML3 and stomatin interact in vesicles as well as at the plasma membrane in living cells.

During the course of live-microscopy experiments, we realized that STOML3- and stomatin-containing vesicles are highly mobile. In both CHO and DRG cells, we observed fast, bidirectional movement of the vesicles interspersed with short stationary phases indicative of an association with the cytoskeleton (see electronic supplementary material, movies S1 and S2). Indeed, co-staining of the microtubule network with tubulin antibodies revealed a close association between STOML3-containing vesicles and microtubules in DRG neurons (figure 1*c*). Furthermore, live-cell imaging of DRG neurons in which fluorescently tagged tubulin end-binding protein-3 (EB3) and STOML3 were expressed revealed movement of STOML3 vesicles along growing microtubules, with vesicles sometimes crossing over from one microtubule bundle to another (see electronic supplementary material, movie S2). We investigated this further by pre-incubating CHO cells with compounds that disrupt the cytoskeleton and tracked vesicular movement over the course of 5 min. Manipulation of actin polymerization (cytochalasinD and jasplakinolide) or stabilization of microtubules with taxol had no effect on the mobility of vesicles, while destabilization of microtubules with nocodazole significantly reduced movement of STOML3 vesicles (figure 1*d*).

In live-cell imaging experiments with DRG neurons transfected with fluorescently tagged STOML3, we tracked moving vesicles and found that their average speed in any given field of view varied between 0.3 and 1.2 μm s^–1^ (mean 0.62 ± 0.02 µm s^–1^; data from 13 neurons, five experiments; figure 1*e*, grey bars). The observed speed of movement is consistent with the speed of kinesin-based movement and with the transport of signalling endosomes in sensory axons [25–28]. Interestingly, we noticed a small sub-population of very long tubular or ‘worm-like’ STOML3 vesicles (defined as greater than 1 µm, eight neurons, four experiments), which moved at a significantly faster rate (mean velocity of 1.59 ± 0.15 µm s^–1^) than the bulk of STOML3 vesicles (unpaired *t*-test *p* < 0.001, red bars in figure 1*e*; figure 1*c* and electronic supplementary material, movie S2). We never observed such fast-moving elongated vesicles in CHO cells transfected with fluorescently tagged STOML3.

We observed tubular STOML3 vesicles moving in a saltatory fashion, which was reminiscent of the movement of mitochondria in sensory axons [29]. We thus used live-cell imaging of sensory neurons transfected with a STOML3-enhanced green fluorescent protein (EGFP) construct and labelled with mitotracker red. The red and green channels were monitored simultaneously using live-cell confocal microscopy, and it was clear that the STOML3 vesicle pool was completely distinct from the red labelled mitochondria (see the electronic supplementary material, movie S3).

### 3.2. Stomatin-like protein-3 interacts with acid-sensing ion channels in vesicles

**Figure 2.**
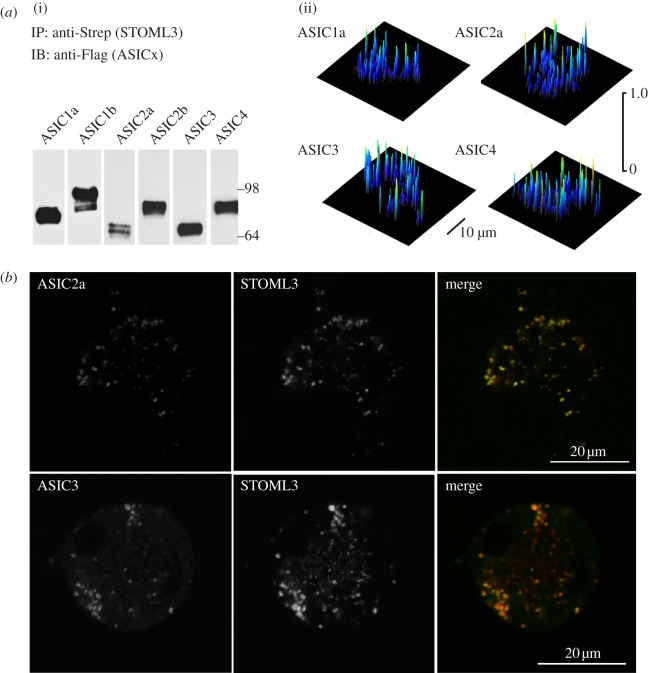
STOML3 interacts with ASICs. (*a*) (i) Flag-tagged ASIC subunits are co-immunoprecipitated by Strep-tagged STOML3 from transiently transfected CHO cells. (ii) Representative surface plots of FRET efficiency of individual cells demonstrating FRET between STOML3 and ASIC subunits. (*b*) STOML3 and ASIC2a and ASIC3 co-localize in DRG neurons.

Stomatin and STOML3 can both modulate the gating of ASICs [9,23]. We therefore carried out co-immunoprecipitation experiments with Strep-tagged STOML3 and Flag-tagged ASICs. We co-immunoprecipitated STOML3 with all six ASIC subunits tested (ASIC1a, 1b, 2a, 2b, 3 and 4) in CHO cells (figure 2*a*). From these experiments, one would predict that ASICs should co-localize with STOML3 in the vesicle pool described earlier. Indeed, we did observe consistent colocalization of STOML3 with ASIC2a and ASIC3 in DRG neurons (figure 2*b*). In addition, in CHO cells, we measured strong FRET signals between STOML3 and ASIC subunits, which were mostly localized to a vesicular compartment (figure 2*a*). The FRET efficiency between STOML3 and ASIC subunits often reached levels as high as 40 per cent (figure 2*a*). The mean, cell-averaged FRET signal between STOML3 and ASICs was similar, approximately 15 per cent, regardless of which ASIC subunit was tested (apparent FRET efficiency: ASIC1a: 14.40 ± 0.51%, ASIC1b: 13.94 ± 0.73%, ASIC2a: 13.42 ± 0.52%, ASIC2b: 13.13 ± 0.49%, ASIC3: 15.52 ± 0.67%, ASIC4: 15.36 ± 0.65%). Thus, although ASICs are integral membrane proteins, another site of interaction with STOML3 is within a mobile vesicle pool in the cytoplasm.

### 3.3. Mapping of the stomatin-like protein-3 interaction domain

Our data suggested that the subcellular distribution of STOML3 and its interaction with stomatin and ASICs might be mutually dependent. We explored this possibility by generating mutant STOML3 constructs and evaluating their sub-cellular localization and capacity to form complexes with stomatin and ASICs. STOML3 has a topology typical of stomatin-like proteins in that it has a short cytoplasmic N-terminus (amino acids 1–19), a hydrophobic region (amino acids 21–50) and a long cytoplasmic C-terminal domain (amino acids 51–287) comprising the stomatin signature domain. We took an unbiased approach and generated large deletions within each of these regions.

**Figure 3.**
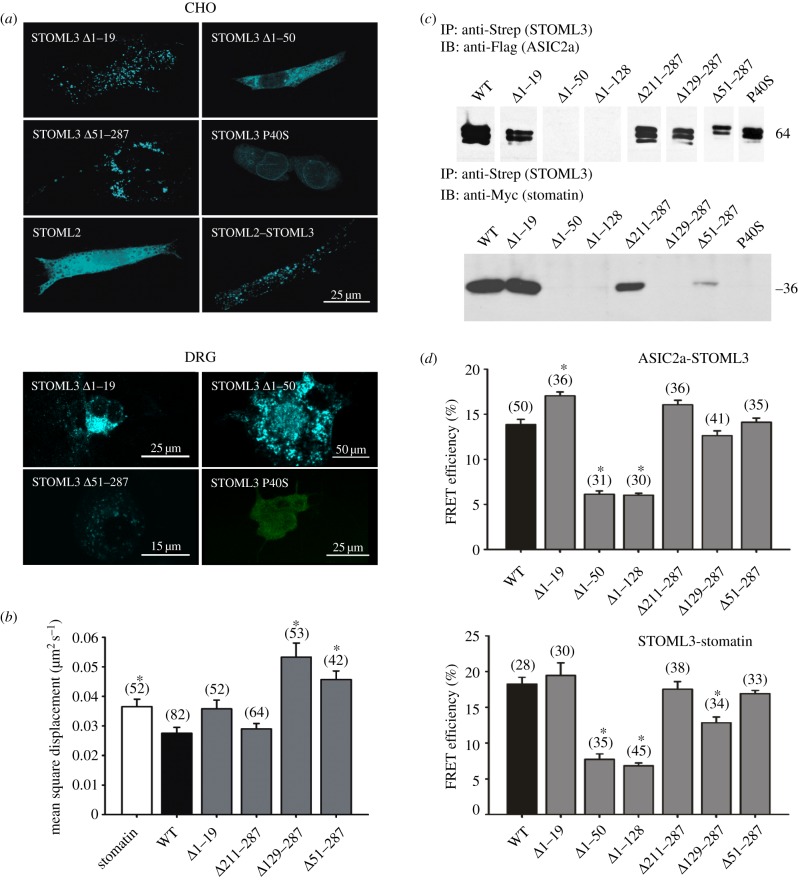
The N-terminal hydrophobic domain of STOML3 is required for localization to vesicles and complex formation. (*a*) Vesicular distribution of STOML3 mutants in CHO cells (top) and DRG neurons (bottom) depends on the integrity of the hydrophobic domain (amino acids 20–50). (*b*) Measurements of STOML3 vesicle velocity in CHO cells. The mean velocity of STOML3 labelled vesicles is shown for wild-type (WT) and STOML3 mutants that localize to vesicles. (*c*,*d*) Strep-tagged STOML3 mutants missing the first 50 or 128 amino acids do not co-immunoprecipitate Flag-tagged ASIC2a (*c*) and the same mutants have significantly lower average FRET efficiency values compared with control (*d*). Mutations in the N- and C-terminus of STOML3 abolish interaction with stomatin as demonstrated by co-immunoprecipitation and FRET experiments. *p < 0.05 versus WT.

We found that complete or partial removal of the N- or C-terminus of STOML3 did not alter the sub-cellular localization of STOML3; CFP-tagged mutants still located to vesicles (figure 3*a*) and were highly mobile (figure 3*b*). However, deletion of the first 50 amino acids (STOML3*Δ*1–50), which includes the hydrophobic domain, resulted in a diffuse cytoplasmic expression of STOML3 (figure 3*a*). Similarly, mutation of a key residue (P40S) that may be required for correct membrane topology of stomatins [30] and cholesterol binding in MEC-2 [14] also abolished the vesicular distribution of STOML3. We explored this finding further by generating a chimaeric construct consisting of the N-terminus and hydrophobic region of STOML3 inserted into another stomatin-like protein, STOML2. Wild-type STOML2 does not contain a hydrophobic domain and was distributed evenly throughout the cytoplasm of CHO cells (figure 3*a*). Strikingly, exchange of this region with STOML3 (amino acids 1–43 from STOML2 with amino acids 1–52 from STOML3) markedly altered its distribution such that the chimaeric protein now localized to vesicles in the same way as STOML3 (figure 3*a*).

Having established that the hydrophobic region of STOML3 is necessary and sufficient for localization in vesicles, we sought to determine whether this region is also required for interaction with stomatin and ASICs. We performed co-immunoprecipitation experiments to identify physical interactions between the proteins (figure 3*c*) and FRET measurements to assess the spatial position of interaction within living cells (figure 3*d*). As expected, deletion of the hydrophobic region of STOML3 and subsequent redistribution away from vesicles abrogated the interaction between STOML3 and stomatin or ASIC2a. However, the point mutation P40S (which abolished vesicular localization of STOML3) also prevented interaction with stomatin, but did not affect immunoprecipitation with ASIC2a (figure 3*c,d*). Similarly, deletion of a central region of the C-terminus of STOML3 (STOML3*Δ*129–287) prevented interaction with stomatin without influencing the localization of STOML3 (figure 3*c,d*).

**Figure 4.**
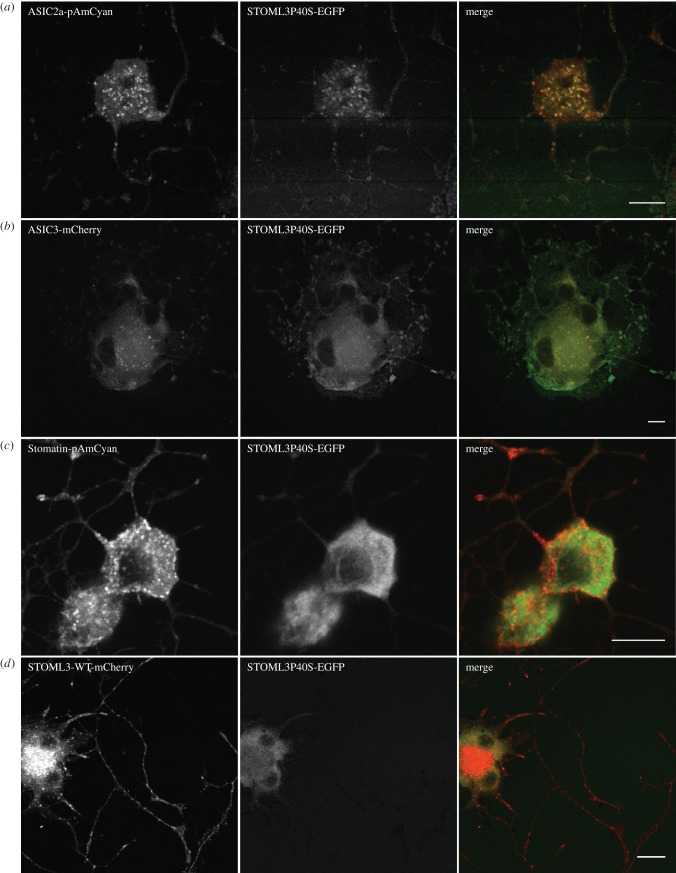
ASIC channels recruit P40S STOML3 mutant protein to vesicles. (*a*) Overexpression of ASIC2a in sensory neurons leads to a relocalization of P40S STOML3 to the ASIC2a-positive vesicular compartment. (*b*) Overexpression of ASIC3 recruits P40S STOML3 to vesicles but to a lesser extent that ASIC2a. (*c*,*d*) Overexpression of stomatin (*c*) or wild-type STOML3 (*d*) does not alter the cytoplasmic localization of P40S STOML3. (*a*–*d*) Scale bars, 10 µm.

The robust physical interaction between the P40S STOML3 mutant protein and ASIC2a was especially puzzling, as these two proteins were localized to different subcellular compartments. We thus asked whether the localization of the P40S STOML3 mutant was altered in sensory neurons that are co-transfected with constructs expressing wild-type ASIC2a, ASIC3, stomatin and STOML3. The results of this analysis, shown in figure 4, indicated that over-expression of ASIC2a in single sensory neurons led to a re-localization of the P40S STOML3 protein to the ASIC2a-positive vesicular compartment (figure 4*a*; 13 cells examined from three independent transfections). A similar phenomenon was observed when sensory neurons were transfected with plasmids encoding fluorescently labelled ASIC3 and P40S STOML3, but here the number of double-positive vesicles was much lower than that observed in cells overexpressing ASIC2a (12 cells from three independent transfections; figure 4*b*). In contrast, in sensory neurons that expressed fluorescently labelled stomatin (eight cells from two independent transfections) or STOML3 (seven cells from two independent transfections) together with P40S STOML3, the protein did not re-localize to the vesicular compartment but remained largely cytoplasmic (figure 4*c,d*). These data provide a potential reason as to why a robust physical interaction was still seen between ASIC2a and the P40S STOML3 protein but not between the same mutant protein and stomatin (figure 3).

Thus, a physical interaction with ASIC2a is dependent upon a vesicular localization and the hydrophobic region of STOML3. The STOML3–stomatin interaction also requires localization to vesicles and an additional region in the C-terminus of the protein. A previous study has shown that residues in the C-terminus of stomatin (amino acid 185 and amino acids 264–272) are responsible for homo-oligomerization of the protein [31], suggesting that this region may be important for assembling higher order structures possibly comprising multiple stomatin-like proteins.

**Figure 5.**
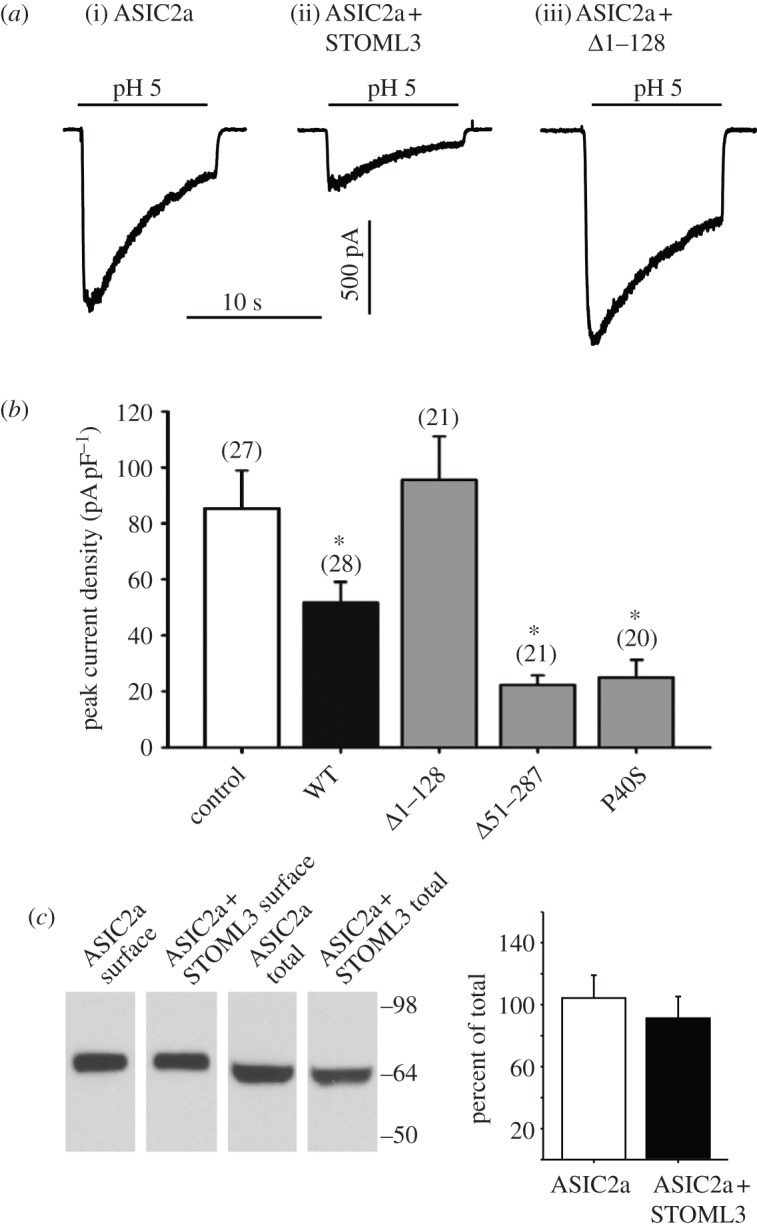
STOML3 modulates acid-gated ASIC2a currents but not surface expression of ASIC2a. (*a*) Examples of inward currents evoked by pH 5 application in CHO cells expressing ASIC2a alone (i), in combination with STOML3 (ii) or in the presence of *Δ*1–128 mutant (iii). (*b*) Mean peak current densities recorded from CHO cells superfused with pH 5 after transfection with ASIC2a and wild-type STOML3 or mutant STOML3 constructs. (*c*) Cell surface and total protein levels of ASIC2a are not altered by co-expression of STOML3, control = ASIC2a, WT = ASIC2a + STOML3, *Δ*1–128 = ASIC2a + STOML3-*Δ*1–128, *Δ*51–287 = ASIC2a + STOML3-*Δ*51–287, P40S = ASIC2a + STOML3-P40S (*n* = 5 separate transfections, *p < 0.05 versus control, *n* = 20–28 tested).

As a physiological measure of the interaction between STOML3 and ASICs, we recorded acid-gated currents in ASIC2a and STOML3 expressing CHO cells using the whole-cell patch-clamp technique. Low pH activates recombinantly expressed ASICs with a rapid, transient current, and stomatin-like proteins are known to regulate the amplitude and kinetics of this current [9,23]. We found that co-expression of STOML3 with ASIC2a significantly reduced the amplitude of the ASIC2a current at pH 5 (figure 5*a,b*), but not with a saturating stimulus of pH 4 (data not shown). No significant effects were observed on the kinetics of the pH-gated current in these experiments (data not shown). Importantly, in agreement with our co-immunoprecipitation and FRET experiments, deletion of the hydrophobic region eliminated the effect of STOML3 on acid-gated currents, while the P40S point mutation and C-terminal deletions behaved similar to wild-type STOML3 (figure 5*a,b*). We performed a similar series of experiments using ASIC3 as an interaction partner of STOML3 and also observed inhibition of the acid-gated current (data not shown).

In the light of the vesicular expression of STOML3, changes in ASIC current amplitude in the presence of STOML3 could result from altered trafficking and membrane expression of ASIC subunits. We investigated this by enriching populations of ASIC2a-YFP and ASIC2a-YFP plus STOML3-CFP cells using fluorescently activated cell sorting and measuring levels of surface biotinylated ASIC2a. Overexpression of STOML3 did not alter ASIC2a surface expression, indicating that the presence of additional STOML3 is not sufficient to alter the balance of ASIC2a subunit membrane turnover (figure 5*c*). Instead, our data point towards a role for STOML3 in directly modulating ASIC2a gating.

### 3.4. Molecular characterization of stomatin-like protein-3-containing vesicles

**Figure 6.**
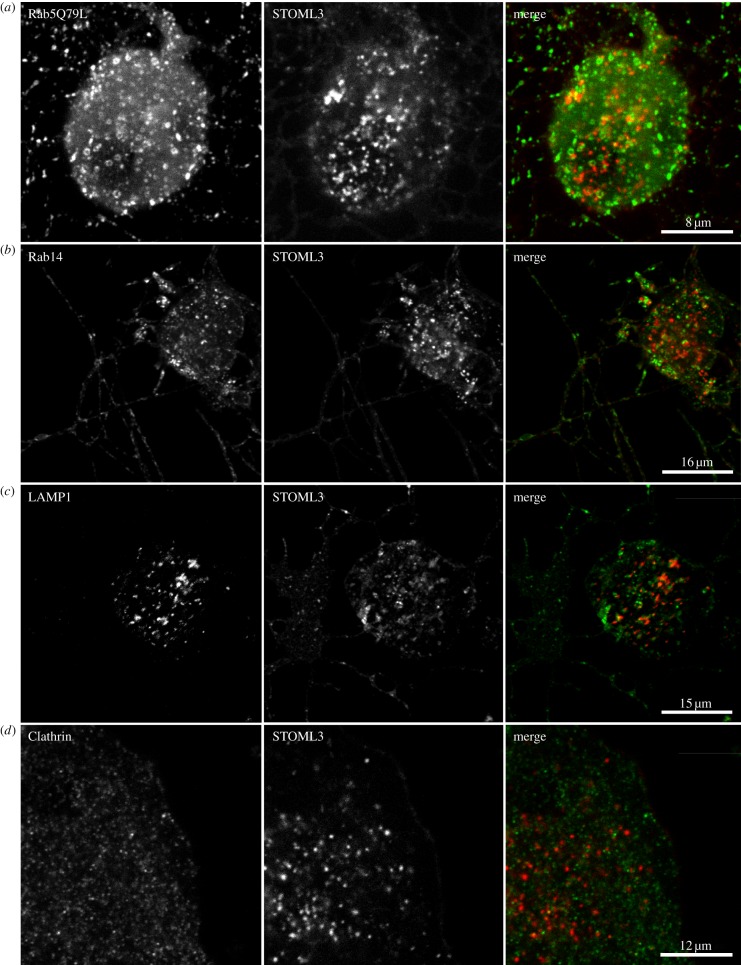
STOML3-containing vesicles are not associated with a classical endocytic vesicle pool. DRG neurons were co-transfected with STOML3-mCherry and either EGFP tagged Rab5 (Q79L mutant) (*a*), Rab14 (*b*) or LAMP1 (*c*), or immunostained for clathrin (*d*). Cells were examined using scanning confocal microscopy and no colocalization was observed with any of the markers (right column).

The trafficking and intracellular sorting of vesicles is a fundamental process common to all cells. Members of the Rab GTPase family of proteins play a major role in orchestrating the correct delivery of cargo proteins, and can also control membrane identity and motility [32]. Thus, to elucidate the nature of STOML3-containing vesicles, we performed immunofluorescence and confocal microscopy on DRG neurons co-transfected with STOML3-mCherry and plasmids encoding Rab proteins that are known to reside in distinct endosomal subsets. Cultured DRG neurons were transfected with plasmids encoding STOML3-mCherry and Rab5, a marker for early endosomes [33,34] or Rab14, which is involved in delivery of cargo from the trans-Golgi network (TGN) to the apical plasma membrane in polarized cells [35]. Using confocal microscopy, we found little or no co-localization between STOML3-containing vesicles and those vesicles containing Rab5 (15 cells examined from two independent transfections) or Rab14 (20 cells examined from two independent transfections; figure 6*a,b*).

We next asked whether the STOML3-positive vesicle compartment corresponds to lysosomes. Co-transfection of plasmids encoding STOML3-mCherry and fluorescently labelled lysosome-associated membrane protein-1 (LAMP1) [36,37] also revealed essentially no overlap (figure 6*c*).

A major molecular pathway for endocytosis involves clathrin coating, and we therefore tested whether STOML3-mCherry vesicles correspond to clathrin-coated vesicles in the cell body and neurites of DRG neurons. Immunostaining for clathrin, which reveals clathrin-coated vesicles in our cultured neurons, revealed virtually no overlap with STOML3-mCherry-positive vesicles (eight cells examined from two independent transfections; figure 6*d*). One proviso attached to this experiment is that clathrin coated endocytic vesicles very quickly lose their clathrin coat once endocytosis is complete. Nevertheless, we also did not observe STOML3-mCherry-positive vesicles positive for clathrin at the plasma membrane (figure 6*d*). No obvious co-localization was observed between recombinantly expressed clathrin and STOML3-mEGFP in experiments in which an mCherry-tagged clathrin expression construct was introduced into cells with STOML3-mEGFP-marked vesicles (data not shown). Thus, our evidence suggests that the integral membrane protein STOML3 is most probably internalized via a clathrin-independent pathway [38].

**Figure 7.**
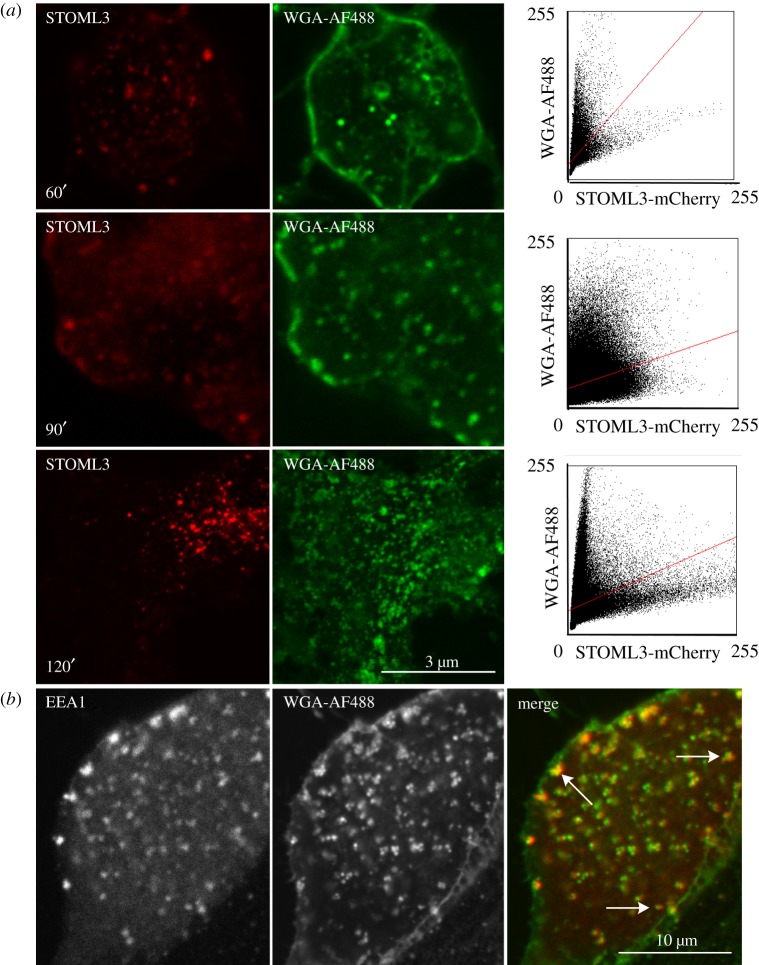
STOML3-containing vesicles locate to a non-endosomal compartment. (*a*) WGA-Alexa Fluor 488 (AF488) labelling of STOML3-mCherry-transfected DRG neurons. Cells were fixed after 60, 90 and 120 min and imaged using a confocal scanning microscope. Co-localization results for all three times are displayed as pixel distribution diagrams (right column). (*b*) In a control experiment, EEA1-mCherry transfected DRG neurons were incubated for 90 min with WGA-AF488 and imaged using a confocal scanning microscope after fixation.

Our data suggested that vesicles containing STOML3 probably do not belong to an early endosomal pool, but we wished to address this question more directly. We thus combined STOML3-mCherry labelling with cell surface labelling using fluorescent wheat germ agglutinin (WGA), as it is known that WGA undergoes fast internalization with subsequent translocation into the TGN [39]. We followed WGA-Alexa Fluor 488-labelled vesicles for 60, 90 and 120 min after the start of incubation (figure 7*a*), but noted no major overlap between STOML3-containing vesicles and WGA-positive endocytic vesicles at any time point (figure 7*a*, upper panel). There was a small amount of overlap between STOML3-mCherry and WGA at 90 min post-incubation, but this was reduced with longer incubation, which may be due to a tendency for WGA to accumulate in the TGN at later time points [39]. Control experiments confirmed that the labelled vesicles in neurons incubated with WGA-Alexa Fluor 488 were largely co-localized with a marker of the early endosomal compartment called EEA1 (figure 7*b*) [40].

**Figure 8.**
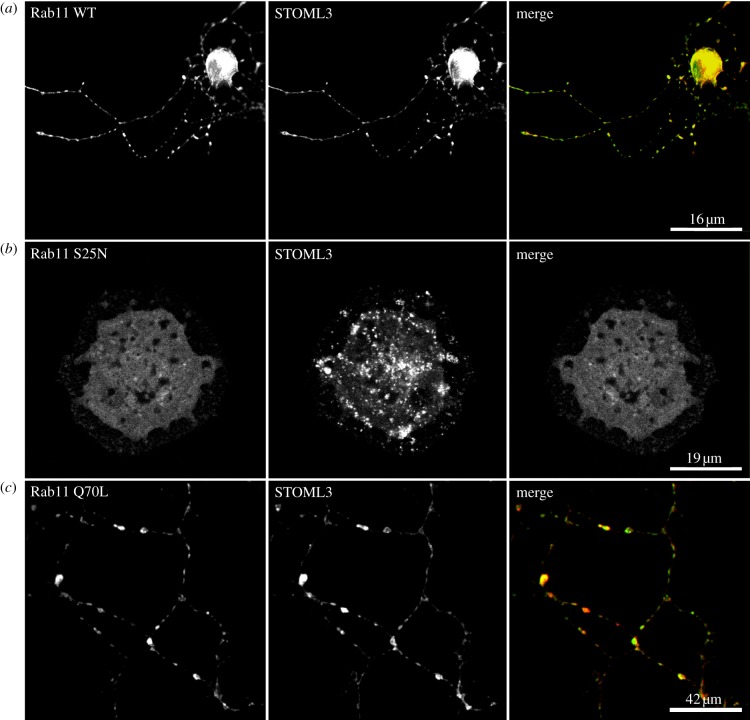
STOML3 colocalizes with Rab11-positive vesicles. (*a*) STOML3-mCherry and Rab11-EGFP exhibit extensive overlap in axons and cell bodies of transfected sensory neurons. (*b*) Transfection of the dominant-negative mutant Rab11 S25N leads to accumulation of STOML3-positive vesicles in the cell body of sensory neurons. (*c*) The constitutively active Rab11 mutant Q70L-EGFP labels large axonal vesicles that are also positive for STOML3-mCherry.

Finally, we considered whether STOML3-containing vesicles might correspond to the recycling endosomal compartment in DRG neurons. We co-transfected plasmids encoding STOML3 and Rab11, a marker of the slow endocytic recycling pathway, and observed pronounced co-localization of these proteins in the same vesicles (20 cells examined from three independent transfections; figure 8*a*). We investigated this further by over-expressing a dominant-negative mutant of Rab11 (S25N) that is locked in the GDP-bound state and not associated with the membrane [41,42]. This led to an accumulation of STOML3 in the cell body and an apparent reduction in trafficking of STOML3-associated vesicles to sensory neuron axons (figure 8*b*). We also examined the constitutively active form of Rab11 (Q70L) and observed characteristic large vesicles in sensory neuron axons that again exhibited prominent overlap with STOML3 (figure 8*c*). Thus, Rab11 serves as a marker for STOML3-containing vesicles in sensory neurons; however, since STOML3 was not detected in other endosomal vesicle compartments, it is possible that these vesicles do not represent classical components of the slow endocytic recycling pathway. Instead, we speculated that they may form a novel class of vesicle with a function in sensory transduction.

**Figure 9.**
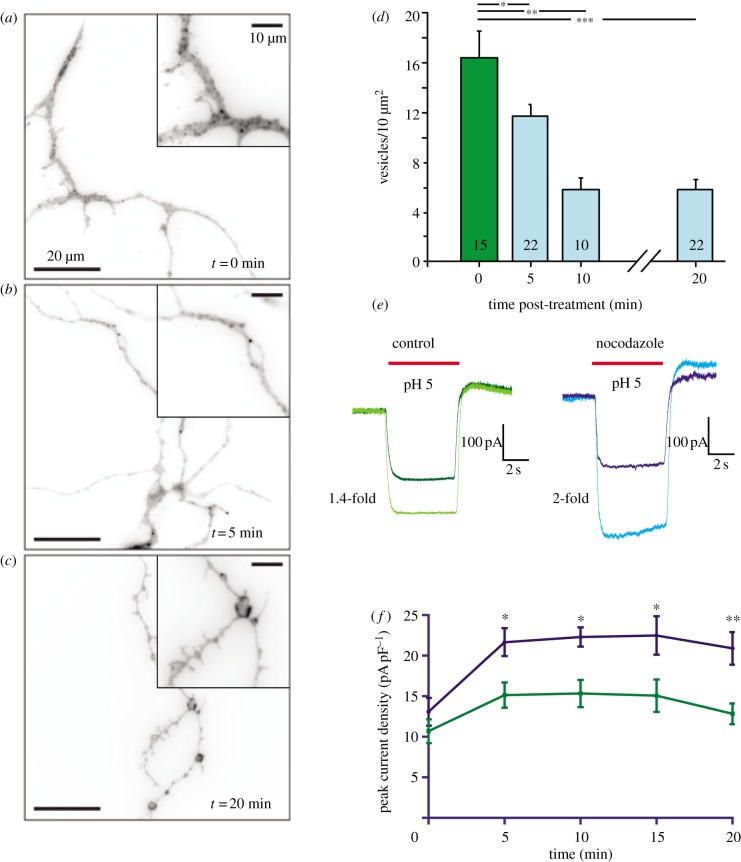
Uncoupling of STOML3 vesicles from microtubules promotes membrane fusion and increases acid-gated currents. (*a–c*) TIRF images (inverted) of STOML3-mCherry in neurites of transfected DRG neurons. Acutely prepared DRG neurons expressing STOML3-mCherry were treated with 32 µM nocodazole for 0 (*a*), 5 (*b*) and 20 min (*c*) and then fixed with 4% PFA. (*d*) The application of nocodazole to the cells resulted in a significant reduction in the number of STOML3-containing vesicles/unit area, after 5 min. (*e*) Example traces showing sustained currents activated by acid (red bars) in DRG neurons under control conditions (dark green, time 0, light green, time 10 min) and in the presence of nocodazole (dark blue, time 0, light blue, time 10 min), demonstrating that current amplitude increases over time, an effect that is larger in the presence of nocodazole. (*f*) Quantification of acid-gated peak current density over time in the presence (blue line, *n* = 10) and absence (green line, *n* = 15) of nocodazole. Acid-gated currents are significantly larger at all time points in the presence of nocodazole, two-way ANOVA; *p < 0.05 and **p < 0.01.

We reasoned that if STOML3 vesicles do indeed have a distinct role in sensory transduction, then they must ultimately fuse with the plasma membrane with consequences for transduction. We had previously observed that nocodazole treatment slowed the movement of STOML3 vesicles, but we also detected a redistribution of STOML3 to the membrane following this treatment. In sensory neurons, nocodazole application primarily disrupts non-stabilized microtubules [43] and we found that this treatment appeared to uncouple STOML3-positive vesicles from microtubules. We used this paradigm in combination with total internal reflection fluorescence microscopy (TIRF) to assess the fusion of STOML3-mCherry-positive vesicles with the plasma membrane (figure 9*a*). TIRF enables the imaging of fluorescent signals in a very thin area, including the plasma membrane and a region less than 200 nm immediately adjacent to the membrane. Here, we acquired TIRF images from fixed cells prior to drug application (0 min), and at time points 5, 10 and 20 min following nocodazole treatment. Prior to treatment, many STOML3-positive vesicles were clearly visible in sensory neuron axons as discrete puncta above the membrane (figure 9*a*). Strikingly, 5 min after nocodazole application, we observed a significant reduction in vesicle number and a redistribution of STOML3 fluorescence to the plasma membrane (figure 9*b*). Membrane fusion appeared to have reached a plateau after 10 min and was accompanied by the formation of varicosities in the neurites, as has been described previously [44] (figure 9*c*). The formation of varicosities was observed only in cells in which STOML3 was overexpressed and not in cells expressing cytoplasmically located GFP (data not shown). Thus, the fusion of STOML3-containing vesicles may lead to physical changes in the plasma membrane, but this may be due to the overexpression of STOML3. In this context, it is probably important that the endogenous STOML3 level is normally quite low in sensory neurons [9].

Vesicle fusion should result in the insertion of vesicular contents such as ASIC subunits, which may be accompanied by an increase in acid-evoked conductance across the membrane. We tested this idea directly by measuring acid-gated currents in sensory neurons in the presence of nocodazole in the recording pipette (figure 9*e*). Repetitive stimulation of sensory neurons with brief applications of pH 5 buffer led to an increase in the magnitude of sustained currents over the course of minutes that was substantially larger in the presence of nocodazole (figure 9*e,f*). Importantly, this effect was not due to an unspecific increment in channel activity because both macroscopic inward and outward voltage-gated currents were not modulated by nocodazole treatment (data not shown). Thus, our data suggest that the highly mobile STOML3 vesicle pool does not correspond to a classical endosomal pathway in primary sensory neurons, but may represent a population of vesicles with a specialized role in sensory transduction. We speculate that this newly characterized STOML3-positive vesicle pool is specialized to deliver transduction components to the membrane. The molecular and physiological characterization of the STOML3-positive vesicle pool described in this paper is the first step in the characterization of a type of vesicle that we propose to name the ‘transducosome’.

## 4. Discussion

Here we demonstrate that mammalian stomatin-family proteins interact directly with each other and with ASIC subunits within a vesicular compartment in primary sensory neurons and in CHO cells. Using biochemical, electrophysiological and imaging techniques, we identify the membrane insertion region in the N-terminus of STOML3 as being required for interaction with ASIC subunits and insertion into vesicles. Furthermore, we suggest that STOML3 defines a novel vesicle pool that has the molecular characteristics of what might be termed a ‘transducosome’.

Stomatin-family proteins, in particular STOML3 and MEC-2, play an essential role in the transduction of mechanical stimuli by sensory neurons in both invertebrates and vertebrates [9–12,16,17,45]. Some members of the ASIC family (e.g. ASIC2 and ASIC3) have also been shown to have a modulatory role in regulating mouse mechanoreceptor sensitivity [21,22], but do not appear to constitute the core mechanosensitive channel in these neurons [46,47]. An initial model of sensory mechanotransduction in *C. elegans* proposed that mechanosensitive ion channels MEC-4 and MEC-10 are anchored between the extracellular matrix and microtubules and that differential displacement of these elements leads to channel gating. MEC-2, a stomatin-like protein, was suggested to tether the channels to microtubule endpoints and thus provide a rigid link between the membrane and the cytoskeleton [48]. More recently, an ultrastructural analysis of the *C. elegans* mechanotransduction complex has revealed that extracellular matrix proteins occupy distinctive cellular domains that are non-overlapping with MEC-2. Furthermore, the mechanosensitive channels do not associate with microtubule endpoints, suggesting that a rigid complex is unlikely to exist in nematode sensory neurons [45]. Moreover, MEC-2 and other stomatin-like proteins have been demonstrated to bind cholesterol and alter the lipid environment of the associated ion channels, supporting a more dynamic role for these proteins [14]. These findings accord well with our observations in mammalian sensory neurons and CHO cells. We detected the majority of STOML3 in a vesicular compartment and our FRET experiments indicated that the physical interactions with stomatin and ASIC subunits are likely to predominate here as well as in the plasma membrane. Although it is clear that ASIC subunits and stomatin can interact, the very high FRET signals found in the vesicle may in part be due to molecular crowding of these over-expressed proteins in the confines of small vesicles. STOML3-containing vesicles also associated with microtubules, a finding that has its precedent in observations made in *C. elegans* where MEC-2 may physically interact with tubulin, but not at the membrane [45].

We investigated the identity of the STOML3-containing vesicles by co-expressing known vesicle markers with STOML3. The size and speed of the vesicles led us to consider that they might belong to an endosomal population. Endosomes have been well characterized in sensory neurons and are known, for example, to be important retrograde signalling components in neurotrophin receptor trafficking [49]. Surprisingly, we found no co-localization of STOML3 with early endosomes, lysosomes or with the Rab14-positive biosynthetic compartment. Similarly, STOML3 vesicles did not appear to be clathrin-coated near to the membrane, indicating that STOML3 is probably internalized via a clathrin-independent pathway. Indeed the only vesicle marker that co-localized with STOML3 was Rab11, which has been shown to be a mediator in the slow endocytic recycling pathway in non-neuronal cell types [50,51]. Interestingly, in neurons, Rab11-positive vesicles have been implicated in the transport of important signalling proteins to the plasma membrane: for example, trafficking of AMPA receptors to dendritic spines [52], axonal targeting of trkA neurotrophin receptors in sympathetic neurons [53] and transport of β1-integrin receptors to the growth cones of adult DRG neurons in culture [54]. Our data suggest that STOML3 vesicles in acutely cultured DRG neurons do not correspond to a classical recycling endosome population, as STOML3 was largely absent from the early part of the endosomal pathway (figure 7). The *in vivo* morphology of DRG neurons is very distinct from any other neuron in that a single pseudounipolar process gives rise to peripheral and central branches that are functionally specialized for transduction and synaptic transmission, respectively. Strikingly, the receptive field of one DRG neuron in the mouse skin is very far removed from the cell body; assuming a cell soma size of 30 µm this axonal distance (approx. 30 mm) is equivalent to 1000 cell diameters. We thus speculate that Rab11/STOML3-positive vesicles in these cells may be specialized to accumulate and transport transduction components in one molecularly distinct compartment from the cell soma to distant transduction sites in the target tissue. Indeed, the association of STOML3-containing vesicles with other transduction molecules such as stomatin and ASICs supports the idea that they might represent a nascent ‘transducosome’ in sensory neurons. Interestingly, Rab11 vesicles in peripheral neurons are mobile and are transported predominantly in the anterograde direction along axons in compartmentalized cultures. Furthermore, the velocity of STOML3 vesicles observed in this study was in agreement with measurements of Rab11 vesicle movement made by others [53,54]. Intriguingly, a sub-population of STOML3 vesicles were elongated and moved significantly faster than smaller STOML3 vesicles in DRG neurons (figure 1*e*); it is however unclear whether such vesicles are functionally distinct from other STOML3-positive vesicles. We have no direct evidence that such long vesicles are Rab11-positive and it is known that Rab11 vesicles move more slowly than non-Rab11 vesicles [53,54]. As well as stomatin-like proteins, we have recently shown that a large extracellular protein is necessary for mechanotransduction in DRG cells [55,56]; it is thus also possible that this filamentous protein is trafficked within the STOML3-positive vesicle pool.

The function of STOML3 within vesicles is difficult to address directly. However, stomatin-family proteins can directly modulate the amplitude of ASIC-mediated currents at the plasma membrane [9,23]. Accordingly, we observed a reduction in the amplitude of acid-gated ASIC currents in the presence of STOML3. The effects of STOML3 overexpression are unlikely to be due to changes in trafficking of ASIC subunits to the membrane as the surface level of ASIC2a was not different between control CHO cells and *STOML3* overexpressing cells. We now demonstrate that truncations of the STOML3 protein that include the hydrophobic membrane insertion domain abolish the ability of the protein to modulate ASICs and also its targeting to vesicles. Furthermore, physical interactions were observed between the P40S STOML3 mutant protein and ASIC2a despite the fact this mutant protein does not normally reside in a vesicular compartment (figures 3 and 4). However, we found that co-expression with ASIC2a in high amounts drives the P40S STOML3 protein to the vesicular compartment so that both modulation of the ASIC current and a physical interaction between these proteins are associated with a vesicular localization. Thus our data are consistent with the idea that there is a functional link between targeting of STOML3 to vesicles and its ability to modulate ASIC-mediated currents. We also found that STOML3-positive vesicles fused to the plasma membrane after disruption of microtubules with nocodazole. This observation allowed us to ask whether the mass fusion of STOML3-positive vesicles to the plasma membrane can lead to an alteration of behaviour of membrane resident ion channels, as would be predicted by the ‘transducosome’ hypothesis. Indeed we did find that acid-gated currents, most likely mediated by a combination of different ASIC subunits, increased substantially in amplitude with the same time course as the observed fusion of STOML3 vesicles monitored with high-resolution TIRF microscopy (figure 9).

In summary, we hypothesize that the function of STOML3 vesicles is to assemble and prime the transduction complex for insertion into the plasma membrane. This would accord well with the cholesterol-binding properties of stomatin-like proteins [14], and one such priming event might be regulation of the membrane lipid environment. Thus, the highly mobile STOML3 vesicles we observed here could be an essential component for the incorporation of functional mechanotransduction complexes into the peripheral terminals of sensory neurons.

## 5. Experimental procedures

### 5.1. Plasmids

The STOML3-EGFP expression plasmid has been described previously and has been shown to retain its biological activity. A red version of STOML3 was generated by replacing EGFP with mCherry red fluorescent protein and a Strep-tagged cyan version by cloning into pAmCyan1-N1 (Clontech). Mouse (1a, 1b, 4) and rat (2a, 2b, 3) full-length ASIC sequences were cloned into pEYFP-C1 (Clontech) and pFLAG-CMV-2 (Sigma). Mouse stomatin was introduced into pEYFP-C1 and pcDNA6-Myc (Invitrogen). All other fluorescently tagged proteins were cloned from mouse DRG-derived cDNA using standard cloning techniques. Mutations of STOML3 were achieved with the ‘QuikChange II XL Site Directed Mutagenesis Kit’ (Stratagene) as described by [57]. The EYFP-AmCyan1 fusion construct was generated by standard cloning techniques, and CFP-TRAF2TRAF-YFP was a kind gift of Dr Liusheng He.

### 5.2. Sensory neuron culture and transfection

For all experiments, isolated DRG neurons from either STOML3^−/−^ mice or wild-type C57/Bl6 animals were cultured on laminin substrates as previously described [9]. Before plating on coverslips or μ-dishes (ibidi GmbH, Germany) for live-cell imaging, freshly prepared neurons were transfected using the Nucleofector system (Amaxa Biosystems). In brief, neurons from one animal were resuspended in 100 µl of Rat Neuron Nucleofector Solution and a total 4–7 µg of plasmid DNA at room temperature. The mixture was transferred to a cuvette and electroporated with the preinstalled program A-033. After electroporation, cells were transferred to 1 ml of RPMI medium and plated on laminin-covered dishes or coverslips.

All immunocytochemistry and live-cell imaging were conducted on cells 24–30 h after transfection.

### 5.3. Live-cell imaging

Three-cube FRET imaging was performed on a Axiovert 200 microscope (Zeiss) equipped with a DUAL-View beam splitter (Optical Insights). The system was calibrated with two fluophore-tandem constructs of differing linker length (EYFP-AmCyan1 and CFP-TRAF2TRAF-YFP) as described by [58]. ImageJ was used for image processing (background subtraction, thresholding), the calculation of FRET efficiency and the unbiased selection of regions of interest for analysis.

For generating BiFC protein pairs, mouse cDNAs for STOML3 or stomatin were cloned into the pBiFC-VN173 or pBiFC-VC155 vectors (kind gift from Dr Chang-Deng Hu, Purdue University, West Lafayette, USA). To detect protein–protein interactions using BiFC, CHO cells were transiently transfected with two plasmids, encoding Venus-fragment tagged bait and prey and incubated for 24 h. Following fixation with 4 per cent PFA, cells were imaged using epifluorescence on an Olympus IX81, with a 40× oil immersion lens.

Vesicle tracking was performed using ImageJ and the Manual Tracking plugin (F.P. Cordelières, Institut Curie, Orsay) for DRG cells or Particle Tracker v. 1.5 [59] for CHO cells. The speed of a single particle was calculated as an average speed in μm s^–1^. When indicated, cells were preincubated for 1 h with 1 µM cytochalasinD (Sigma), jasplakinolide (Calbiochem), nocodazole (Sigma) or taxol (Sigma) and the drugs were also present during experiments.

### 5.4. Coimmunoprecipitation and immunoblotting

CHO cells were transiently transfected with two epitope-tagged constructs (STOML3-Strep with either ASICx-Flag or stomatin-Myc, 1 : 1) using FugeneHD (Roche). After 48 h, cells were lysed with RIPA buffer (50 mM Tris (pH 7.4), 300 mM NaCl, 1mM EDTA, 0.5% deoxycholic acid, 0.5% Triton-X100, 0.1% SDS, 1 mM DTT and a protease inhibitor cocktail (Sigma)) and incubated with anti-Strep antibody (Novagen) and Protein G agarose beads (Roche) overnight. After rigorous washing, samples were subjected to SDS-PAGE and co-precipitated proteins detected by immunoblotting with anti-Flag (Stratagene) or anti-Myc (Roche) antibody, as indicated.

### 5.5. Cell surface labelling

CHO cells were transfected with ASIC2a-YFP and ASIC2a-YFP plus STOML3-CFP plasmids. Positively transfected cells were enriched using a three laser standard configuration FACS Aria (BD Biosciences) cell sorter (100 µm nozzle, 20 psi). Cell surface ASIC2a-YFP was biotinylated and isolated by means of the commercially available ‘Pierce Cell Surface Protein Isolation Kit’ according to the manufacturer's instructions. Half of the protein extracted from CHO cells expressing either ASIC2a alone or in combination with STOML3 was not purified but kept as internal control of total protein load and compared with the surface fraction on immunoblots stained with anti-GFP (Roche).

### 5.6. Electrophysiology

For CHO cell experiments, whole-cell patch clamp recordings were carried out at room temperature with an EPC-10 patch clamp amplifier (HEKA Elektronic, Germany). Solution-filled, borosilicate glass pipettes (110 mM KCl, 10 mM NaCl, 1 mM MgCl_2_, 1 mM EGTA, and 10 mM HEPES, adjusted to pH 7.3 with KOH) had a resistance of 3–4 M*Ω*. The extracellular solution consisted of 140 mM NaCl, 4 mM KCl, 2 mM CaCl_2_, 1 mM MgCl_2_, 4 mM glucose and 10 mM HEPES (pH 7.4 with NaOH). For pH 5 and pH 4 solutions, HEPES was replaced by MES. Transiently transfected CHO cells were held at −60 mV and superfused with low pH solutions for 10 s. The resulting peak current amplitudes (normalized for cell capacitance) were analysed with Pulse and FitMaster software (HEKA).

For DRG neurons, whole-cell, patch clamp recordings were conducted at room temperature within 24 h of dissection, using the following solutions: extracellular (in mM)—NaCl (140), KCl (4), CaCl_2_ (2), MgCl_2_ (1), glucose (4), HEPES (10), adjusted to pH 7.4 or pH 5.0 with NaOH and HCl as appropriate; intracellular—KCl (110), NaCl (10), MgCl_2_ (1), EGTA (1) and HEPES (10), adjusted to pH 7.3 with KOH. Patch pipettes were pulled from borosilicate glass capillaries (Hilgenberg) and had a resistance of 3–6 M*Ω*. Recordings were made using an EPC-9 amplifier (HEKA) and PatchMaster software (HEKA). Based upon our observation that STOML3 is predominantly present in large-diameter DRG neurons (Lapatsina & Lewin 2010, unpublished data), we made recordings from large diameter sensory neurons (more than 28 µm diameter). Cells were held at −60 mV and whole-cell currents were recorded at 20 kHz, pipette and membrane capacitance were compensated using PatchMaster macros and series resistance was compensated by approximately 70 per cent. Cells were constantly perfused with a pH 7.4 solution, and immediately after going whole cell a standard voltage-step protocol was performed whereby cells were held at −120 mV for 150 ms before stepping to the test potential (from −80 to +50 mV in 5 mV increments) for 40 ms, returning to the holding potential (−60 mV) for 200 ms between sweeps. Immediately thereafter, a 5 s pulse of pH 5.0 solution was applied, a procedure that was repeated after 5, 10, 15 and 20 min along with a repetition of the voltage-step protocol. To test the effects of nocodazole on voltage- and acid-activated currents, a 16 mM stock solution was dissolved in the intracellular solution to give a final concentration of 32 µM. The peak amplitude of acid- and voltage-gated currents was measured using FitMaster software (HEKA). Our analysis focused on sustained currents because these were found to be the most common (approx. 60% in both conditions) compared with the very variable (in terms of both kinetics and amplitude) transient currents that were observed. Two-way ANOVAs examining the effects of nocodazole upon peak current amplitude were conducted in Prizm (GraphPad Software, Inc.).

### 5.7. Immunofluorescence staining, confocal fluorescence imaging and data processing

For immunocytochemistry, cultivated DRG neurons were fixed in 4 per cent paraformaldehyde in PBS (pH 7.4) for 10–15 min at room temperature, washed in PBS and subsequently permeabilized with 0.05 per cent Triton-X for 10 min at room temperature. Non-specific binding was blocked by incubating the culture in 3 per cent goat serum at 37°C for 30 min. Incubations with primary antibodies were carried out overnight at 4°C or for 45 min at 37°C in 3 per cent goat serum. Subsequently, coverslips were washed twice with PBS and incubated with a fluorescent secondary antibody for 45 min at 37°C. After several washes, coverslips were mounted and examined on a Leica SP5 confocal microscope.

### 5.8. Wheat germ agglutinin labelling and quantification

For WGA labelling, DRG neurons, transiently transfected for 24 h before the experiment with a wild-type STOML3-mCherry construct, were incubated at 37°C with 30 μg ml^–1^ WGA-Alexa Fluor 488 (Invitrogen) in HBSS with 0.1 mM Ca^2+^, 1 mM Mg^2+^ and 1 per cent bovine serum albumin for either 60, 90 or 120 min. Thereafter, the cells were washed to remove unbound labelled WGA and fixed with 4 per cent paraformaldehyde for subsequent analysis of co-localization of WGA with the STOML3 compartment and with markers of endocytic compartments.

To estimate the degree of WGA-Alexa Fluor 488—STOML3-mCherry colocalization, high-resolution images of transfected WGA labelled neurons were acquired and grey values of two pictures were plotted against each other using ImageJ v. 1.42q, JACoP plugin [60]. Results were displayed in a pixel distribution diagram (scattered plot), where the intensity of a given pixel in the green image was used as the *x*-coordinate of the scattered plot and the intensity of the corresponding pixel in the red image as the *y*-coordinate. Using this kind of analysis, a complete colocalization will result in a pixel distribution along a straight line whose slope will depend on the fluorescent ratio between the two channels, in a partial colocalization event the pixel distribution will be off the axes, and in a case of exclusive staining the pixel intensities will be distributed along the axes of the scatter plot.

### 5.9. Total internal reflection fluorescence microscopy

DRG cells expressing STOML3-mCherry were cultured in eight-well µ-slides (ibidi GmbH, Germany) coated with laminin. After 24 h of growth DRGs had established neurite trees and robust expression of STOML3-mCherry. Microtubules were disrupted by adding nocodazole to the media at a final concentration of either 16 µM or 32 µM. Cells were fixed at various time points between 0 and 30 min post addition of nocodazole, using 4 per cent PFA in PBS (15 min, room temperature). In parallel experiments in non-transfected DRG neurons, microtubules were labelled using a mouse monoclonal anti-acetylated tubulin antibody (clone 6-11B-1, Sigma Aldrich) and rat monoclonal anti-tyrosinated tubulin antibody (clone YL1/2, Abcam) to distinguish stabilized from non-stabilized microtubules, respectively. Primary antibodies were used at a dilution of 1/1000 and subsequently labelled with goat anti-mouse-Alexa Fluor 488 and goat anti-rat-Alexa Fluor 633 antibodies (Invitrogen), both used at a dilution of 1/1000.

Samples were imaged using TIRF microscopy using an Olympus TIRFM system, with a 100×, 1.45 NA, TIRF objective and laser illumination using a 488 nm Argon laser and a 543 nm He–Ne laser.

## Supplementary Material

Supplementary Figure 1

## References

[1] Tavernarakis N Driscoll M Kyrpides NC 1999 The SPFH domain: implicated in regulating targeted protein turnover in stomatins and other membrane-associated proteins. Trends Biochem. Sci. 24, 425–427 10.1016/S0968-0004(99)01467-X (doi:10.1016/S0968-0004(99)01467-X) 10542406

[2] Lapatsina L Brand J Poole K Daumke O Lewin GR 2012 Stomatin-domain proteins. Eur. J. Cell Biol. 91, 240–245 10.1016/j.ejcb.2011.01.018 (doi:10.1016/j.ejcb.2011.01.018) 21501885

[3] Green JB Young JP 2008 Slipins: ancient origin, duplication and diversification of the stomatin protein family. BMC Evol. Biol. 8, 44 10.1186/1471-2148-8-44 (doi:10.1186/1471-2148-8-44) 18267007 PMC2258279

[4] Rivera-Milla E Stuermer CA Malaga-Trillo E 2006 Ancient origin of reggie (flotillin), reggie-like, and other lipid-raft proteins: convergent evolution of the SPFH domain. Cell Mol. Life Sci. 63, 343–357 10.1007/s00018-005-5434-3 (doi:10.1007/s00018-005-5434-3) 16389450 PMC11135987

[5] Yokoyama H Fujii S Matsui I 2008 Crystal structure of a core domain of stomatin from *Pyrococcus horikoshii* illustrates a novel trimeric and coiled-coil fold. J. Mol. Biol. 376, 868–878 10.1016/j.jmb.2007.12.024 (doi:10.1016/j.jmb.2007.12.024) 18182167

[6] Kobayakawa K Hayashi R Morita K Miyamichi K Oka Y Tsuboi A Sakano H 2002 Stomatin-related olfactory protein, SRO, specifically expressed in the murine olfactory sensory neurons. J. Neurosci. 22, 5931–5937 12122055 10.1523/JNEUROSCI.22-14-05931.2002 PMC6757947

[7] Goldstein BJ Kulaga HM Reed RR 2003 Cloning and characterization of SLP3: a novel member of the stomatin family expressed by olfactory receptor neurons. J. Assoc. Res. Otolaryngol. 4, 74–82 10.1007/s10162-002-2039-5 (doi:10.1007/s10162-002-2039-5) 12239636 PMC3202447

[8] Tadenev AL Kulaga HM May-Simera HL Kelley MW Katsanis N Reed RR 2011 Loss of Bardet-Biedl syndrome protein-8 (BBS8) perturbs olfactory function, protein localization, and axon targeting. Proc. Natl Acad. Sci. USA 108, 10 320–10 325 10.1073/pnas.1016531108 (doi:10.1073/pnas.1016531108) PMC3121838 21646512

[9] Wetzel C 2007 A stomatin-domain protein essential for touch sensation in the mouse. Nature 445, 206–209 10.1038/nature05394 (doi:10.1038/nature05394) 17167420

[10] O'Hagan R Chalfie M Goodman MB 2005 The MEC-4 DEG/ENaC channel of *Caenorhabditis elegans* touch receptor neurons transduces mechanical signals. Nat. Neurosci. 8, 43–50 10.1038/nn1362 (doi:10.1038/nn1362) 15580270

[11] Martinez-Salgado C 2007 Stomatin and sensory neuron mechanotransduction. J. Neurophysiol. 98, 3802–3808 10.1152/jn.00860.2007 (doi:10.1152/jn.00860.2007) 17942620

[12] Huang M Gu G Ferguson EL Chalfie M 1995 A stomatin-like protein necessary for mechanosensation in *C. elegans*. Nature 378, 292–295 10.1038/378292a0 (doi:10.1038/378292a0) 7477350

[13] Boute N 2000 NPHS2, encoding the glomerular protein podocin, is mutated in autosomal recessive steroid-resistant nephrotic syndrome. Nat. Genet. 24, 349–354 10.1038/74166 (doi:10.1038/74166) 10742096

[14] Huber TB 2006 Podocin and MEC-2 bind cholesterol to regulate the activity of associated ion channels. Proc. Natl Acad. Sci. USA 103, 17 079–17 086 10.1073/pnas.0607465103 (doi:10.1073/pnas.0607465103) PMC1859892 17079490

[15] Mannsfeldt AG Carroll P Stucky CL Lewin GR 1999 Stomatin, a MEC-2 like protein, is expressed by mammalian sensory neurons. Mol. Cell Neurosci. 13, 391–404 10.1006/mcne.1999.0761 (doi:10.1006/mcne.1999.0761) 10383825

[16] Brown AL Liao Z Goodman MB 2008 MEC-2 and MEC-6 in the *Caenorhabditis elegans* sensory mechanotransduction complex: auxiliary subunits that enable channel activity. J. Gen. Physiol. 131, 605–616 10.1085/jgp.200709910 (doi:10.1085/jgp.200709910) 18504316 PMC2391253

[17] Goodman MB Ernstrom GG Chelur DS O'Hagan R Yao CA Chalfie M. 2002 MEC-2 regulates *C. elegans* DEG/ENaC channels needed for mechanosensation. Nature 415, 1039–1042 10.1038/4151039a (doi:10.1038/4151039a) 11875573

[18] Zhang S 2004 MEC-2 is recruited to the putative mechanosensory complex in *C. elegans* touch receptor neurons through its stomatin-like domain. Curr. Biol. 14, 1888–1896 10.1016/j.cub.2004.10.030 (doi:10.1016/j.cub.2004.10.030) 15530389

[19] Molliver DC Immke DC Fierro L Pare M Rice FL McCleskey, EW 2005 ASIC3, an acid-sensing ion channel, is expressed in metaboreceptive sensory neurons. Mol. Pain 1, 35 10.1186/1744-8069-1-35 (doi:10.1186/1744-8069-1-35) 16305749 PMC1308857

[20] Page AJ Brierley SM Martin CM Hughes PA Blackshaw LA 2007 Acid sensing ion channels 2 and 3 are required for inhibition of visceral nociceptors by benzamil. Pain 133, 150–160 10.1016/j.pain.2007.03.019 (doi:10.1016/j.pain.2007.03.019) 17467171

[21] Price MP 2000 The mammalian sodium channel BNC1 is required for normal touch sensation. Nature 407, 1007–1011 10.1038/35039512 (doi:10.1038/35039512) 11069180

[22] Price MP 2001 The DRASIC cation channel contributes to the detection of cutaneous touch and acid stimuli in mice. Neuron 32, 1071–1083 10.1016/S0896-6273(01)00547-5 (doi:10.1016/S0896-6273(01)00547-5) 11754838

[23] Price MP Thompson RJ Eshcol JO Wemmie JA Benson CJ 2004 Stomatin modulates gating of acid-sensing ion channels. J. Biol. Chem. 279, 53 886–53 891 10.1074/jbc.M407708200 (doi:10.1074/jbc.M407708200) 15471860

[24] Hu CD Kerppola TK 2003 Simultaneous visualization of multiple protein interactions in living cells using multicolor fluorescence complementation analysis. Nat. Biotechnol. 21, 539–545 10.1038/nbt816 (doi:10.1038/nbt816) 12692560 PMC1820765

[25] Cai D Verhey KJ Meyhofer E 2007 Tracking single Kinesin molecules in the cytoplasm of mammalian cells. Biophys. J. 92, 4137–4144 10.1529/biophysj.106.100206 (doi:10.1529/biophysj.106.100206) 17400704 PMC1877757

[26] Gagliano J Walb M Blaker B Macosko JC Holzwarth G 2009 Kinesin velocity increases with the number of motors pulling against viscoelastic drag. Eur. Biophys. J. 39, 801–813 10.1007/s00249-009-0560-8 (doi:10.1007/s00249-009-0560-8) 19921171

[27] Shtridelman Y Holzwarth GM Bauer CT Gassman NR DeWitt DA Macosko JC. 2009 *In vivo* multimotor force–velocity curves by tracking and sizing sub-diffraction limited vesicles. Cell. Mol. Bioeng. 2, 190–199 10.1007/s12195-009-0064-8 (doi:10.1007/s12195-009-0064-8)

[28] Cui B Wu C Chen L Ramirez A Bearer EL Li W-P Mobley WC Chu S. 2007 One at a time, live tracking of NGF axonal transport using quantum dots. Proc. Natl Acad. Sci. USA 104, 13 666–13 671 10.1073/pnas.07061921044 (doi:10.1073/pnas.07061921044) PMC1959439 17698956

[29] Morris RL Hollenbeck PJ 1993 The regulation of bidirectional mitochondrial transport is coordinated with axonal outgrowth. J. Cell. Sci. 104, 917–927 8314882 10.1242/jcs.104.3.917

[30] Kadurin I Huber S Grunder S 2009 A single conserved proline residue determines the membrane topology of stomatin. Biochem. J. 418, 587–594 10.1042/BJ20081662 (doi:10.1042/BJ20081662) 19032151

[31] Umlauf E Mairhofer M Prohaska R 2006 Characterization of the stomatin domain involved in homo-oligomerization and lipid raft association. J. Biol. Chem. 281, 23 349–23 356 10.1074/jbc.M513720200 (doi:10.1074/jbc.M513720200) 16766530

[32] Stenmark H 2009 Rab GTPases as coordinators of vesicle traffic. Nat. Rev. Mol. Cell Biol. 10, 513–525 10.1038/nrm2728 (doi:10.1038/nrm2728) 19603039

[33] Rubino M Miaczynska M Lippe R Zerial M 2000 Selective membrane recruitment of EEA1 suggests a role in directional transport of clathrin-coated vesicles to early endosomes. J. Biol. Chem. 275, 3745–3748 10.1074/jbc.275.6.3745 (doi:10.1074/jbc.275.6.3745) 10660521

[34] Gorvel JP Chavrier P Zerial M Gruenberg J 1991 rab5 controls early endosome fusion *in vitro*. Cell 64, 915–925 10.1016/0092-8674(91)90316-Q (doi:10.1016/0092-8674(91)90316-Q) 1900457

[35] Kitt KN Hernandez-Deviez D Ballantyne SD Spiliotis ET Casanova JE Wilson JM 2008 Rab14 regulates apical targeting in polarized epithelial cells. Traffic 9, 1218–1231 10.1111/j.1600-0854.2008.00752.x (doi:10.1111/j.1600-0854.2008.00752.x) 18429929 PMC2773667

[36] Barriocanal JG Bonifacino JS Yuan L Sandoval IV 1986 Biosynthesis, glycosylation, movement through the Golgi system, and transport to lysosomes by an N-linked carbohydrate-independent mechanism of three lysosomal integral membrane proteins. J. Biol. Chem. 261, 16 755–16 763 3782140

[37] Lewis V Green SA Marsh M 1985 Glycoproteins of the lysosomal membrane. J. Cell Biol. 100, 1839–1847 10.1083/jcb.100.6.1839 (doi:10.1083/jcb.100.6.1839) 3922993 PMC2113609

[38] Gong Q Huntsman C Ma D 2008 Clathrin-independent internalization and recycling. J. Cell Mol. Med. 12, 126–144 10.1111/j.1582-4934.2007.00148.x (doi:10.1111/j.1582-4934.2007.00148.x) 18039352 PMC3823476

[39] Vetterlein M Ellinger A Neumuller J Pavelka M 2002 Golgi apparatus and TGN during endocytosis. Histochem. Cell Biol. 117, 143–150 10.1007/s00418-001-0371-1 (doi:10.1007/s00418-001-0371-1) 11935290

[40] Simonsen A 1998 EEA1 links PI(3)K function to Rab5 regulation of endosome fusion. Nature 394, 494–498 10.1038/28879 (doi:10.1038/28879) 9697774

[41] Ullrich O Reinsch S Urbe S Zerial M Parton RG 1996 Rab11 regulates recycling through the pericentriolar recycling endosome. J. Cell Biol. 135, 913–924 10.1083/jcb.135.4.913 (doi:10.1083/jcb.135.4.913) 8922376 PMC2133374

[42] Schlierf B Fey GH Hauber J Hocke GM Rosorius O 2000 Rab11b is essential for recycling of transferrin to the plasma membrane. Exp. Cell Res. 259, 257–265 10.1006/excr.2000.4947 (doi:10.1006/excr.2000.4947) 10942597

[43] Fukushima N Furuta D Hidaka Y Moriyama R Tsujiuchi T 2009 Post-translational modifications of tubulin in the nervous system. J. Neurochem. 109, 683–693 10.1111/j.1471-4159.2009.06013.x (doi:10.1111/j.1471-4159.2009.06013.x) 19250341

[44] Jacobs JR Stevens JK 1986 Experimental modification of PC12 neurite shape with the microtubule-depolymerizing drug Nocodazole: a serial electron microscopic study of neurite shape control. J. Cell Biol. 103, 907–915 10.1083/jcb.103.3.907 (doi:10.1083/jcb.103.3.907) 3745274 PMC2114310

[45] Cueva JG Mulholland A Goodman MB 2007 Nanoscale organization of the MEC-4 DEG/ENaC sensory mechanotransduction channel in *Caenorhabditis elegans* touch receptor neurons. J. Neurosci. 27, 14089–14098 10.1523/JNEUROSCI.4179-07.2007 (doi:10.1523/JNEUROSCI.4179-07.2007) 18094248 PMC6673530

[46] Drew LJ Rohrer DK Price MP Blaver KE Cockayne DA Cesara P Wood JN 2004 Acid-sensing ion channels ASIC2 and ASIC3 do not contribute to mechanically activated currents in mammalian sensory neurones. J. Physiol. 556, 691–710 10.1113/jphysiol.2003.058693 (doi:10.1113/jphysiol.2003.058693) 14990679 PMC1664992

[47] Lechner SG Frenzel H Wang R Lewin GR 2009 Developmental waves of mechanosensitivity acquisition in sensory neuron subtypes during embryonic development. EMBO J. 28, 1479–1491 10.1038/emboj.2009.73 (doi:10.1038/emboj.2009.73) 19322198 PMC2664657

[48] Ernstrom GG Chalfie M 2002 Genetics of sensory mechanotransduction. Annu. Rev. Genet. 36, 411–453 10.1146/annurev.genet.36.061802.101708 (doi:10.1146/annurev.genet.36.061802.101708) 12429699

[49] Howe CL Valletta JS Rusnak AS Mobley WC 2001 NGF signaling from clathrin-coated vesicles: evidence that signaling endosomes serve as a platform for the Ras-MAPK pathway. Neuron 32, 801–814 10.1016/S0896-6273(01)00526-8 (doi:10.1016/S0896-6273(01)00526-8) 11738027

[50] Casanova JE Wang X Kumar R Bhartur SG Navarre J Woodrum JE Altschuler Y Ray GS Goldenring JR 1999 Association of Rab25 and Rab11a with the apical recycling system of polarized Madin-Darby canine kidney cells. Mol. Biol. Cell 10, 47–61 9880326 10.1091/mbc.10.1.47 PMC25153

[51] Horgan CP McCaffrey MW 2009 The dynamic Rab11-FIPs. Biochem. Soc. Trans. 37, 1032–1036 10.1042/BST0371032 (doi:10.1042/BST0371032) 19754446

[52] Correia SS Bassani S Brown TC Lise MF Backos DS El-Husseini A Passafaro M Esteban JA 2008 Motor protein-dependent transport of AMPA receptors into spines during long-term potentiation. Nat. Neurosci. 11, 457–466 10.1038/nn2063 (doi:10.1038/nn2063) 18311135

[53] Ascano M Richmond A Borden P Kuruvilla R 2009 Axonal targeting of Trk receptors via transcytosis regulates sensitivity to neurotrophin responses. J. Neurosci. 29, 11 674–11 685 10.1523/JNEUROSCI.1542-09.2009 (doi:10.1523/JNEUROSCI.1542-09.2009) PMC2775807 19759314

[54] Eva R Dassie E Caswell PT Dick G ffrench-Constant C Norman JC Fawcett JW. 2010 Rab11 and its effector Rab coupling protein contribute to the trafficking of beta 1 integrins during axon growth in adult dorsal root ganglion neurons and PC12 cells. J. Neurosci. 30, 11 654–11 669 10.1523/JNEUROSCI.2425-10.2010 (doi:10.1523/JNEUROSCI.2425-10.2010) PMC6633432 20810886

[55] Hu J Chiang LY Koch M Lewin GR 2010 Evidence for a protein tether involved in somatic touch. EMBO J. 29, 855–867 10.1038/emboj.2009.398 (doi:10.1038/emboj.2009.398) 20075867 PMC2810375

[56] Chiang LY Poole K Oliveira BE Duarte N Sierra YA Bruckner-Tuderman L Koch M Hu J Lewin GR 2011 Laminin-332 coordinates mechanotransduction and growth cone bifurcation in sensory neurons. Nat. Neurosci. 14, 993–1000 10.1038/nn.2873 (doi:10.1038/nn.2873) 21725315

[57] Makarova O Kamberov E Margolis B 2000 Generation of deletion and point mutations with one primer in a single cloning step. Biotechniques 29, 970–972 11084856 10.2144/00295bm08

[58] Chen H Puhl HL III Koushik SV Vogel SS Ikeda SR 2006 Measurement of FRET efficiency and ratio of donor to acceptor concentration in living cells. Biophys. J. 91, L39–L41 10.1529/biophysj.106.088773 (doi:10.1529/biophysj.106.088773) 16815904 PMC1544280

[59] Sbalzarini IF Koumoutsakos P 2005 Feature point tracking and trajectory analysis for video imaging in cell biology. J. Struct. Biol. 151, 182–195 10.1016/j.jsb.2005.06.002 (doi:10.1016/j.jsb.2005.06.002) 16043363

[60] Bolte S Cordelieres FP 2006 A guided tour into subcellular colocalization analysis in light microscopy. J. Microsc. 224, 213–232 10.1111/j.1365-2818.2006.01706.x (doi:10.1111/j.1365-2818.2006.01706.x) 17210054

