# Supplementary figures and images for: Regulation of ASIC channels by a stomatin/STOML3 complex located in a mobile vesicle pool in sensory neurons
Source: Open Biol. 2012 Jun;2(6):120096. doi: 10.1098/rsob.120096 (PMC3390797; doi:10.1098/rsob.120096)

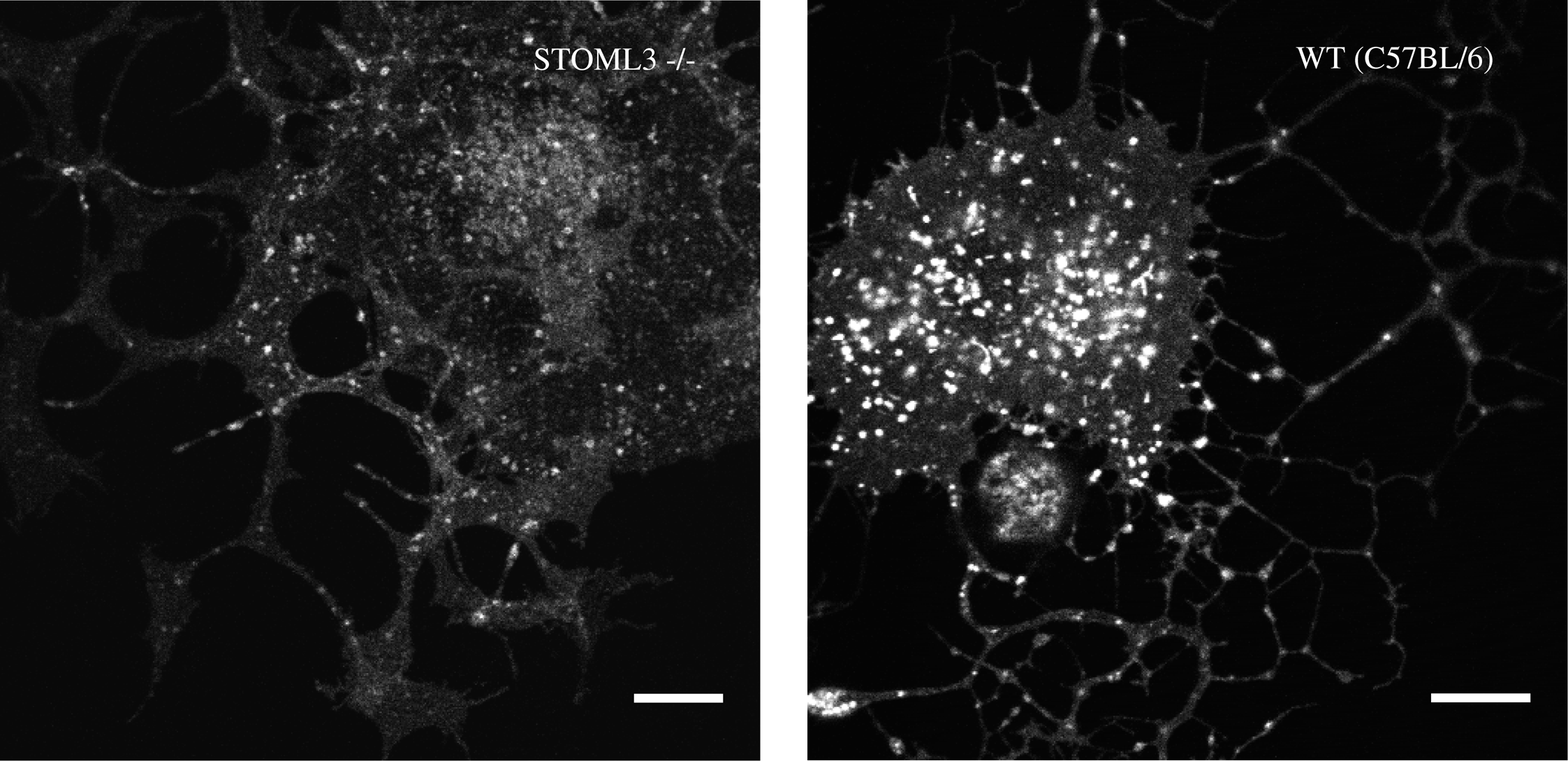

Supplement: Supplementary Figure 1 [file rsob120096-s1.tif]
